# Vascular endothelial growth factor induces the migration of human airway smooth muscle cells by activating the RhoA/ROCK pathway

**DOI:** 10.1186/s12890-023-02803-y

**Published:** 2023-12-13

**Authors:** Chengtian Lv, Yuwen Huang, Ruirong Yan, Yuanmei Gao

**Affiliations:** Department of Pulmonary and Critical Care Medicine; Guangdong Provincial Key Laboratory of Major Obstetric Diseases, Guangdong Provincial Clinical Research Center for Obstetrics and Gynecology, The Third Affiliated Hospital of Guangzhou Medical University, Guangzhou, China

**Keywords:** Vascular endothelial growth factor, RhoA/ROCK pathway, Airway smooth muscle cells, Cell migration, Airway remodeling

## Abstract

**Background:**

Airway remodeling due to increased airway smooth muscle cell (ASMC) mass, likely due to enhanced proliferation, hypertrophy, and migration, has been proven to be highly correlated with decreased lung function in asthma patients. Vascular endothelial growth factor (VEGF) mediates vascular and extravascular remodeling and inflammation and has been proven to be involved in the progression of asthma. Previous studies have focused on the effects of VEGF on ASMC proliferation, but few researchers have focused on the effects of VEGF on human ASMC migration. The purpose of this study was to explore the effect of VEGF on the migration of ASMCs and its related signaling pathway mechanism to provide evidence for the treatment of airway remodeling.

**Methods:**

We examined the effects of VEGF induction on ASMC migration and explored the mechanisms involved in ASMC migration.

**Results:**

We found by wound healing and Transwell assays that VEGF promoted ASMC migration. Through the Cell Counting Kit-8 (CCK-8) experiment, we found that VEGF had no significant effect on the proliferation of ASMCs, which excluded the involvement of cell proliferation in the process of wound healing. Moreover, a cellular immunofluorescence assay showed that VEGF promoted F-actin reorganization, and Western blotting showed that VEGF improved RhoA activation and myosin phosphatase targeting subunit-1 (MYPT_1_) and myosin light chain (MLC) phosphorylation in ASMCs. Treatment with the ROCK inhibitor Y27632 significantly attenuated the effects of VEGF on MYPT_1_/MLC activation and cell migration.

**Conclusion:**

In conclusion, the results suggest that the promigratory function of VEGF activates the RhoA/ROCK pathway, induces F-actin reorganization, improves the migration of ASMCs, and provides a better rationale for targeting the RhoA/ROCK pathway for therapeutic approaches in airway remodeling.

**Supplementary Information:**

The online version contains supplementary material available at 10.1186/s12890-023-02803-y.

## Introduction

Asthma is one of the most common noncommunicable chronic lung diseases and threatens the health of 334 million people worldwide [1]. Airway remodeling, which is one of the pathological features of asthma, can cause narrowing of the lumen and restriction of airflow, leading to reduced lung function in asthmatic patients. Previous studies have shown that airway remodeling is not only common in adults with asthma but also found in children with refractory asthma [2]. At present, bronchodilators and glucocorticoids are the main drugs used to treat asthma, but these drugs cannot effectively inhibit or reverse airway remodeling [3, 4]. Airway smooth muscle cells (ASMCs) are the structural cells of the airway wall, and a key structural change in airway remodeling is an increase in ASMC mass. For many years, it was generally believed that the increase in ASMC mass is due to the proliferation and hypertrophy of ASMCs, which is related to the severity of asthma and the decline in lung function [5–7]. Gizycki et al. found that the number of submucosal ASMCs in asthmatic patients was increased by lung tissue biopsy within 24 hours after allergen stimulation, and this reaction was faster than the time of the ASMC proliferation cycle [8]. Therefore, they believed that the migration of airway smooth muscle cells to the submucosal layer was the potential mechanism of airway remodeling in asthma. Therefore, further exploration of novel potential mechanisms that underlie ASMC migration during airway remodeling is necessary for the development of new therapeutic methods to reverse airway remodeling.

Vascular endothelial growth factor (VEGF) is a multifunctional regulator of angiogenesis that promotes the formation of new blood vessels, increases the permeability of blood vessels, and promotes the migration and proliferation of vascular endothelial cells. In animal experiments, mice treated with VEGF exhibit asthma-like phenotypes, such as increased lung inflammation, vascular remodeling, tissue edema, increased smooth muscle cell mass, and airway hyperresponsiveness [8]. ASAI K et al. showed that VEGF contents are increased in the sputum of asthmatic patients, and the level of VEGF is negatively correlated with the diameter of the trachea [9–11]. Therefore, VEGF, which is a mediator of vascular and extravascular remodeling and inflammation, has been confirmed to participate in the development of asthma. Qing-Mei Pei et al. showed that VEGF enhances the expression of ADAM-33 and the proliferation of ASMCs by activating the VEGFR2/ERK1/2 signaling pathway [12]. Snigdha Banerjee et al. showed that VEGF can activate the neurociliarin-1-VEGFR_1_-PI3K axis in human aortic smooth muscle cells and promote migration [13]. Oliver A Stone et al. have also shown that endogenous VEGF exerts a recruitment effect on vascular smooth muscle [14]. However, few researchers have focused on the effects of VEGF on human ASMC migration through the RhoA/ROCK pathway.

Abnormal migration of ASMCs plays an important role in airway remodeling. In complex individual environments, cell migration is controlled by several factors [15]. Previous studies have shown that Rho GTPase family members, including Rho, Rac and Cdc42, control multiple processes, such as cell adhesion, proliferation, contraction and migration [16]. RhoA is a member of the small GTPase Rho family, and RhoA-associated kinase (ROCK) is the downstream effector of RhoA [17]. The RhoA/ROCK signaling pathway activates the downstream protein ROCK through the binding of Rho and GTP, which in turn leads to phosphorylation of the downstream substrate of ROCK, causes cytoskeleton rearrangement, induces actin filament stability and actin myosin contraction, connects the actin network and myosin fibers, and regulates microtubule dynamics, ultimately affecting cell migration [18–21]. In general, cell migration is a coordinated process that involves cell signaling, cytoskeletal changes, biochemical pathways and physical movement. Because the RhoA/ROCK signaling pathway is closely related to cell migration, it is very important to study the signal transduction of the RhoA/ROCK pathway in ASMCs for the treatment of airway remodeling.

Based on the above, we propose the hypothesis that VEGF induces the migration of human ASMCs by activating the RhoA/ROCK signaling pathway.

## Materials and methods

### Primary cell sources and cell culture

The study was conducted according to the guidelines of the Declaration of Helsinki and approved by the institutional review board of the Third Affiliated Hospital of Guangzhou Medical University, with ethics approval ID [2022] No. 009. We obtained tracheal tissues from 4 organ donors, ages 32, 32, 55, and 58, none of whom had underlying disease before death, and informed consent was obtained from all subjects and/or their legal guardians before the study. The smooth muscle tissue was separated from other tracheal tissues, the airway smooth muscle tissue was cut into pieces, and the primary ASMCs were obtained by tissue patch culture [22].

ASMCs were cultured in DMEM/F12 medium supplemented with 10% fetal bovine serum (FBS) and incubated in a cell incubator at 37 °C in 5% CO_2_. Primary ASMCs were used at passages 3–6. Fetal bovine serum contains many nutrients needed for cell growth, including a variety of cell growth factors and hormones. Before the experiment, the ASMC medium was changed to DMEM/F12 without FBS, and the ASMCs were starved for 24 hours. After the addition of VEGF (40 ng.ml^−1^) (PrimeGene Bio-Tech, China), the cells were incubated in a cell incubator at 37 °C in 5% CO_2_. In the inhibition group, Y27632 (10 nM.ml^−1^) (MedChemExpress, China), which is a ROCK_1/2_ target inhibitor, was added to the cells and incubated for 30 minutes before VEGF was added. The concentration of Y27632 stock solution was 10 mM.ml^−1^, and the solvent of the stock solution was dimethyl sulfoxide (DMSO). The concentration of Y27632 needed for the inhibitor group experiment was 10 nM.ml^−1^. During the experiment, the Y27632 stock solution was diluted 10^−6^ times. Therefore, the final concentration of DMSO in the inhibitor group was 0.0001% vol/vol. To maintain consistency in the experimental conditions, we added DMSO at the same volume concentration as the inhibitor group to both the control and experimental groups.

### Wound healing assay

To evaluate the effect of VEGF on ASMC migration, we conducted a wound healing assay. We seeded ASMCs in 6-well cell culture plates, and the cells were allowed to adhere to the well and grow to greater than 90% confluence. Then, the medium was replaced with serum-free medium, and the cells were starved for 24 hours. A 200-μl pipette tip was used to generate a wound in the cell monolayer that was perpendicular to the bottom line, and the dissociated cells were gently removed by washing with PBS (phosphate buffered sodium). Then, serum-free basic medium (DMEM/F12 without FBS and inhibitors) was added to the control group, and DMEM/F12 supplemented with VEGF (40 ng.mL^−1^) was added to the experimental group. The inhibitor group (VEGF+Y27632) (Y27632: 10 nM.mL^−1^) was pretreated with a signaling pathway inhibitor (Y27632) for 30 minutes, and then VEGF was added. The cells were incubated in a cell incubator at 37 °C in 5% CO_2_ for 24 hours. The changes in the wound area were observed under a microscope and photographed at 0, 12 and 24 hours. We used ImageJ software to measure the area of the wound at each time point, compared the area with that observed at 0 hours, and analyzed the migration distance of each group.

### Cell proliferation assessment

We tested the proliferation of ASMCs via the Cell Counting Kit-8 (CCK-8) assay to determine whether cell proliferation was involved in the wound repair process, as previously described [22]. In this study, cells were divided into the control group and experimental (VEGF: 40 ng.ml^−1^) group, and the cells were seeded in 96-well test plates at a density of 1*10^4^ cells per well. After the cells adhered to the well, the medium was replaced with serum-free medium, and the cells were incubated in a cell incubator at 37 °C in 5% CO_2_. After 24 hours of cell starvation, basic medium was added to the control group, and 20, 40 or 80 ng·ml^−1^ VEGF was added to the VEGF groups. After another 24 hours of incubation in the cell incubator, the cells were incubated with 100 μl medium supplemented with a 10% CCK-8 working solution (Genview, China) at 37 °C in 5% CO_2_ for 4 hours. The OD values of each group were measured at a wavelength of 450 nm by an automatic enzyme labeling instrument.

### Transwell assay

To investigate the effect of VEGF on the transmembrane migration of ASMCs, we conducted a Transwell assay. The cells were incubated in basal medium without FBS for 24 hours before the experiment. Then, the cells were digested, resuspended, and seeded in the upper chamber (Corning, USA) at a concentration of 8.0 X 10^5^ cells.ml^−1^ in a total of 200 μl. Medium with or without supplemental VEGF (40 ng.ml^−1^) was added to the lower chamber. The inhibitor group (VEGF+Y27632) (Y27632: 10 nM.mL^−1^) was pretreated with a signaling pathway inhibitor for 30 minutes, and then VEGF was added. The plates were incubated at 37 °C in 5% CO_2_ for 24 hours. We fixed the cells with 4% paraformaldehyde for 15 minutes and then stained the ASMCs with 1% crystal violet for 30 minutes. After washing, the cells on the upper surface of the membrane were gently removed, and then, the cells that had migrated to the lower surface of the membrane were observed and photographed with an inverted microscope (magnification of 200x). ImageJ software was used to count the number of migrated cells.

### Cell immunofluorescence assay

To observe the changes in the cytoskeleton after treatment of ASMCs with VEGF, we conducted a cellular immunofluorescence assay. A total of 5.0 × 10^3^ cells per well were seeded into a 12-well cell culture plate with cover glass on the bottom. After the cells had completely attached to the well, the medium was replaced with basic medium without FBS. After the cells were starved for 24 hours, medium with or without VEGF (40 ng.ml^−1^) was added, PDGF 10 ng.ml^−1^ was added to the positive control group, Y27632 (10 nM.ml^−1^) was added to the inhibitor group, and the cells were incubated at 37 °C in 5% CO_2_ for 12 hours. We washed the cells with PBS three times and fixed the cells with 4% formaldehyde at room temperature for 15 minutes. The cells were washed again with PBS, and then 0.5% Triton X-100 was added and incubated for 10 minutes. A phalloidin-Alexa Fluor 488 (1:150, Solarbio Science & Technology, China) peptide working solution was used to stain the cells at room temperature for 30 minutes. The nuclei were stained with a small amount of DAPI (1:1000 dilution), the glass was sealed with resin, and images were captured under laser confocal imaging (magnification of 600x) and analyzed. The procedure was conducted carefully to minimize the impact of nonspecific stimuli on the cytoskeleton.

### Western blotting analysis

The expression levels of downstream target proteins of the RhoA/ROCK signaling pathway were analyzed by Western blotting. RIPA lysis buffer supplemented with 2% phenylmethanesulfonyl fluoride (PMSF) and 2% protease complex inhibitors was used to lyse the cells on ice, and the protein was collected from the cells. A bicinchoninic acid (BCA) kit (Beyotime Biotechnology, China) was used to measure the protein concentration. We chose β-actin as an internal reference. The molecular weights of ROCK_1_, myosin phosphatase targeting subunit-1 (MYPT_1_), β-actin, and myosin light chain (MLC) were 160 KD, 140 KD, 42 KD, and 18 KD, respectively, so we chose to separate ROCK_1_, MYPT_1_ and β-actin using sodium dodecyl sulfate–polyacrylamide gel electrophoresis (SDS–PAGE) electrophoresis at a concentration of 8% and MLC and β-actin using SDS–PAGE electrophoresis at a concentration of 12%. The proteins were then transferred to polyvinylidene difluoride (PVDF) membranes, which were blocked with 5% skim milk powder at room temperature. The blots were cut prior to hybridization with antibodies and then incubated overnight at 4 °C with antibodies against the following proteins: ROCK_1_ (1:1000, Abcam, UK), MYPT_1_ (D6C1, 1:1000, Cell Signaling Technology, USA), phospho-MYPT_1_ (p-MYPT_1_) (Thr696, 1:1000, Cell Signaling Technology, USA), MLC (D18E2, 1:1000, Cell Signaling Technology, USA), phospho-MLC (p-MLC) (Ser19, 1:1000, Cell Signaling Technology, USA) and β-actin (1:4000, Abbkine, China). The PVDF membranes were washed with TBST (TBS solution containing 0.1% Tween-20) 3 times for 5 minutes each. We then incubated the membranes with the secondary antibody at room temperature for 60 minutes and washed the PVDF membranes with TBST again. The immunoblots were evaluated by a gel imaging system. β-Actin was used as a loading control. Image Lab and ImageJ software were used to analyze the Western blotting results.

### Statistical analysis

The data from each group were analyzed using GraphPad Prism 5.01 software (GraphPad Software, San Diego California, USA). Measurement data are expressed as the mean ± standard deviation (x ± SD). One-way analysis of variance (ANOVA) was used to compare the data when the variance was homogeneous, and the Student-Newman–Keuls-q (SNK-q) test was used for pairwise comparisons. When the variances were uneven, the Kruskal–Wallis H test was used for comparison. When *P* < 0.05, the difference was considered statistically significant.

## Results

### Evaluation of VEGF-induced ASMC migration by wound healing assay

**Fig. 1:**
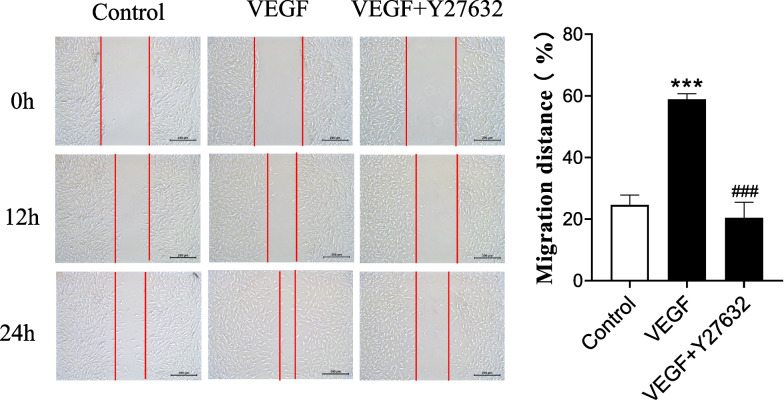
Effects of VEGF on ASMC wound healing. Representative images of the control group, VEGF (40 ng.ml^−1^) group and VEGF (40 ng.ml^−1^) + Y27632 (10 nM.ml^−1^) group at 0 h, 12 h and 24 h after injury. The range marked between the two red lines is the blank area after removing the cells with the tip of the pipette. The histogram compares standardized migration distances at 24 h after injury in the different groups (*n* = 3 independent experiments; ***P < 0.001 vs. the control group, ###*P* < 0.001 vs. the VEGF group; the data are presented as the mean ± SD)

We used a wound healing assay to assess the effect of VEGF on ASMC migration. After incubation with VEGF (40 ng.ml^−1^) for 24 hours, the standard migration distance of the experimental (VEGF) group was increased compared with that of the control group, and the difference was significant (*P* < 0.05). When the cells in the inhibitor group were pretreated with Y27632 (10 nM.ml^−1^) for 30 minutes, the standard migration distance of the VEGF + Y27632 group was decreased compared with that of the VEGF group, and the difference between the groups was significant (*P* < 0.05) (Fig. 1). Taken together, our data suggest that VEGF enhances ASMC migration.

### Effects of VEGF on ASMC proliferation

**Fig. 2:**
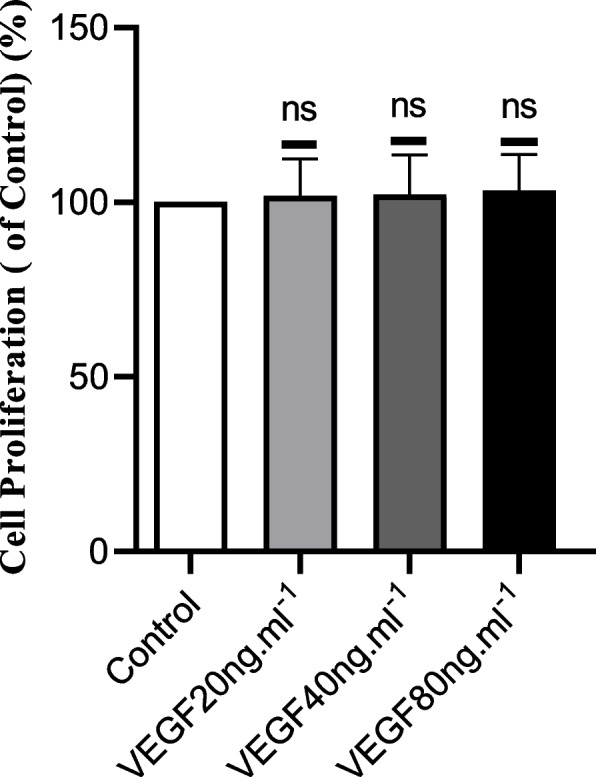
Effect of VEGF on the proliferation of ASMCs. Cell Counting Kit-8 (CCK-8) assays were used to assess the proliferation of ASMCs after incubation with or without VEGF (20, 40 or 80 ng·ml^−1^) for 24 h. The control group was compared with the 40 ng.ml^−1^ VEGF groups. The data are presented as the mean ± SD. *n* = 3 independent experiments; NS indicates no significant difference

We tested the proliferation of ASMCs via the Cell Counting Kit-8 (CCK-8) assay to determine if cell proliferation was involved in the wound repair process. After ASMCs were incubated with VEGF (20, 40 or 80 ng·ml^−1^) for 24 hours, there was no significant difference in cell proliferation between the experimental (VEGF) group and the control group. Therefore, based on the experimental conditions, VEGF at a concentration of 20, 40 or 80 ng·ml^−1^ had no significant effect on ASMC proliferation, which excluded the involvement of cell proliferation in the process of wound repair. As shown in Fig. 2.

### Evaluation of VEGF-induced ASMC migration by Transwell assay

**Fig. 3:**
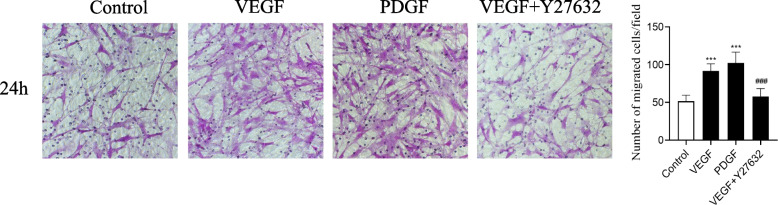
Effects of VEGF on ASMC migration in Transwell assays. Representative images of the control group, VEGF group, PDGF group, and VEGF + Y27632 group after 24 h of incubation with or without VEGF (40 ng.ml^−1^) or Y27632 (10 nM.ml^−1^). The PDGF (10 ng.ml^−1^) group was a positive control group. The number of migrated cells was compared between groups (***P < 0.001 vs. the control group, ###*P* < 0.001 vs. the VEGF group)

We used a Transwell assay to evaluate the effect of VEGF on ASMC migration. We found that the number of cells that had migrated at 24 hours was increased in the 40 ng.ml^−1^ VEGF groups compared with the control group, and the difference was significant (*P* < 0.05). The PDGF group was a positive control group. When the VEGF + Y27632 group was pretreated with Y27632 (10 nM.ml^−1^) for 30 minutes, the number of cells that had migrated in the VEGF + Y27632 group was decreased compared with that in the VEGF group, and the difference was significant (P < 0.05), as shown in Fig. 3. Therefore, our data suggest that Y27632 can inhibit VEGF-induced ASMC migration.

### Effect of VEGF on the cytoskeleton of ASMCs

**Fig. 4:**
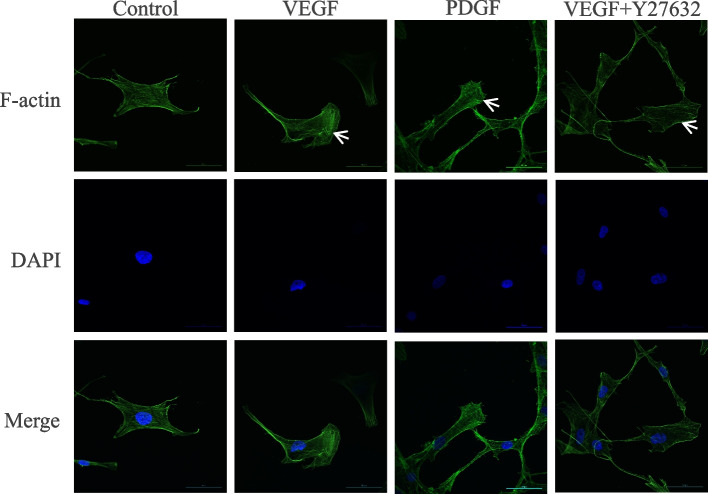
VEGF induces cytoskeletal changes in ASMCs. Polymerized actin in ASMCs treated with VEGF (40 ng.ml^−1^) or PDGF (10 ng.ml^−1^) for 24 h was analyzed by phalloidin staining. ASMCs were preconditioned with Y27632 (10 nM.ml^−1^) for 30 min and then treated with VEGF+ Y27632 for 24 h; then, polymerized actin was analyzed by phalloidin staining. The arrows in the figure show the representative changes in cell morphology in this group

We stained ASMCs with phalloidin Alexa Fluor H 488 to observe the effect of VEGF on the cytoskeleton of migrating ASMCs. Actin exists in both free and polymerized states in cells, namely, G-actin and F-actin. Phalloidin, a toxic cyclic heptad extracted from mushroom phalloidin, specifically binds to F-actin but not G-actin. Phalloidin Alexa Fluor H 488 specifically binds to polymerized F-actin. Under confocal laser scanning microscopy, fluorescently labeled phalloidin clearly showed the morphology and distribution of the cytoskeleton. In the control group, there was no obvious extension of lamellipodia, and a relatively uniform distribution of F-actin was observed under a confocal laser microscope. In the VEGF group, the cell actin filaments increased and polarized, pseudopodia were prominent, and the cytoskeleton was reconstructed. The PDGF group served as the positive control group, and the changes in the cell morphology of the VEGF group and PDGF group were similar. In the VEGF+Y27632 group, the F-actin filament decreased, the distribution was disordered, there were no cell pseudopodia, and cytoskeletal remodeling was significantly inhibited. As shown in Fig. 4. In this study, cytoskeletal remodeling after VEGF stimulation of ASMCs promoted cell migration, and the ROCK inhibitor Y27632 inhibited this migration.

### VEGF increases the phosphorylation of myosin phosphatase targeting subunit-1 (MYPT_1_) and myosin light chain (MLC), activates the RhoA/ROCK pathway, and promotes the migration of ASMCs

**Fig. 5:**
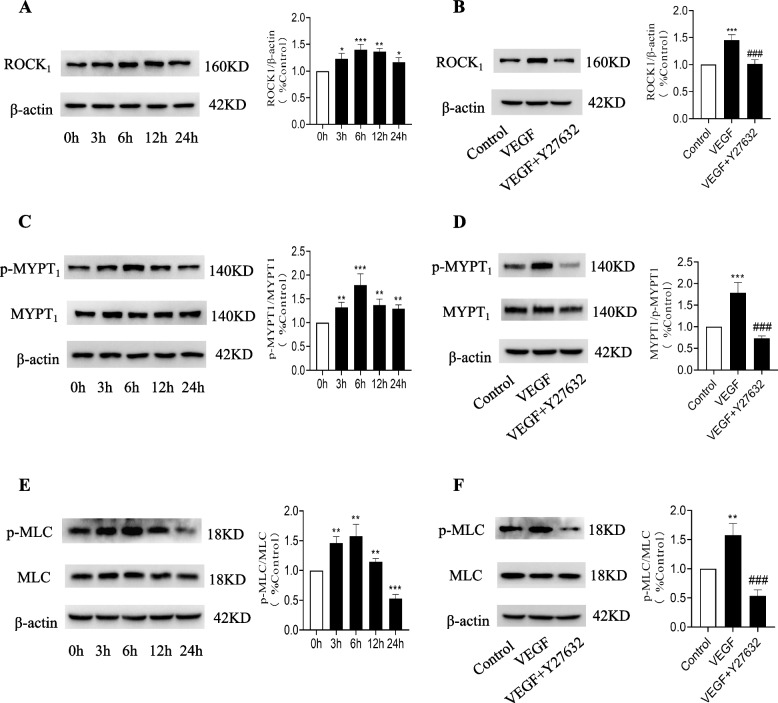
Effects of VEGF on the expression of RhoA-associated kinase 1 (ROCK_1_) (160 KD), phospho-myosin phosphatase targeting subunit-1 (p-MYPT_1_) (140 KD), and phospho-myosin light chain (p-MLC) (18 KD) in ASMCs. **A** Western blotting images of ROCK_1_ expression in ASMCs after 0 h, 3 h, 6 h, 12 h, and 24 h of incubation with VEGF (40 ng.ml^−1^). **B** ASMCs were incubated with VEGF in the presence of Y27632 (10 nM) for 6 hours, and the expression of ROCK_1_ was measured. **C** Western blotting images of MYPT_1_ (140 KD) phosphorylation in ASMCs after 0 h, 3 h, 6 h, 12 h, and 24 h of incubation with VEGF. **D** ASMCs were incubated with VEGF (40 ng.ml^−1^) in the presence of Y27632 for 6 hours, and the phosphorylation of MYPT_1_ was measured. **E** Western blotting images of MLC (18 KD) phosphorylation in ASMCs after 0 h, 3 h, 6 h, 12 h, and 24 h of incubation with VEGF. **F** ASMCs were incubated with VEGF in the presence of Y27632 for 6 hours, and the phosphorylation of MLC was measured. The signal in each lane was quantified by using ImageJ software, and the ratios of p-MYPT_1_/MYPT_1_ expression to that in the control group were calculated (*n* = 3 independent experiments, *P < 0.05, **P < 0.01, ***P < 0.001 vs. the control group; #*P* < 0.05, ##*P* < 0.01, ###*P* < 0.001 vs. the VEGF group, NS vs. the control group, indicates no significant difference)

Cell migration is driven by signaling molecules and is influenced by internal biochemical pathways. The activation of RhoA increases the intracellular level of phosphorylated myosin, promotes the interaction between actin and myosin, and regulates cell contraction and migration. To clarify the trend of phosphorylation of ROCK1, MYPT1, and MLC within 24 hours after VEGF incubation, Western blot analysis was also performed at 24 hours. We used Western blotting to detect the expression of ROCK1, MYPT1, and MLC phosphorylation in ASMCs at five time points after incubation with ASMCs and VEGF for 0 h, 3 h, 6 h, 12 h and 24 h. As shown in Fig. 5 (A), (C), and (E), we observed increased expression levels of ROCK_1_, p-MYPT_1_ (phosphorylation of myosin phosphatase targeting subunit-1) and p-MLC (phosphorylation of myosin light chain) in ASMCs after VEGF treatment by Western blotting. Therefore, our data suggest that VEGF induces ASMC migration by activating the RhoA/ROCK signaling pathway. As shown in Fig. 5 (B), (D), and (F), Y27632 is an inhibitor of ROCK. According to Western blotting analysis, the expression levels of ROCK_1_, p-MYPT_1_ and p-MLC were significantly reduced when Y27632 was added to the VEGF group. Therefore, our data suggest that VEGF activates the RhoA/ROCK_1_/MYPT_1_/MLC cascade in ASMCs, thus promoting ASMC migration by activating the RhoA/ROCK pathway through the increased phosphorylation of its components.

## Discussion

Airway remodeling is one of the pathological characteristics of asthma, and the relevant mechanism has not yet been clarified. In recent years, there have been an increasing number of studies on airway remodeling in asthma. The purpose of this study was to explore the effect of VEGF on ASMC migration. First, we found that VEGF induced ASMCs to promote migration. Second, we further observed that cytoskeletal remodeling promotes migration. Finally, VEGF activates the RhoA/ROCK/MYPT_1_/MLC cascade in ASMCs, thus promoting ASMC migration by activating the RhoA/ROCK pathway through the increased phosphorylation of its components. Treatment with the ROCK inhibitor Y27632 significantly attenuated the effects of VEGF on MYPT_1_/MLC activation and cell migration. Taken together, these data indicated that VEGF activates the RhoA/ROCK pathway, induces F-actin reorganization, and improves the migration of ASMCs.

VEGF was initially defined as a vascular permeability factor. It is a specific growth factor released by tumor cells in the hypoxic environment found by Senger et al. in a study of animal tumor growth [23]. Its role can increase vascular permeability. Meyer N and other researchers found that VEGF is a strong inducer of endothelial cells, which can induce angiogenesis by promoting the proliferation and differentiation of endothelial cells, leading to an increase in airway vascular density and vascular area in asthma, and is the key regulatory factor of airway vascular growth in asthma patients [11]. There are a variety of growth factors and inflammatory mediators in the lungs of asthma patients. These growth factors and inflammatory mediators can promote the migration of ASMCs, including platelet-derived growth factor (PDGF) and transforming growth factor-β (TGF-β), leukotriene, prostaglandin D2 (PGD2), eosinophil chemokine, interleukin-8 (IL-8) and thymic stromal lymphopoietin (TSLP), and these factors play an important role in airway remodeling [24]. VEGF mediates vascular and extravascular remodeling and inflammation.

Our results showed that VEGF promoted the migration of human primary ASMCs in vitro. This suggests that VEGF plays a positive role in promoting airway remodeling in patients with asthma. Previous studies have shown that VEGF exposure has a proliferative effect on ASMCs [25–27]. Our research findings seem to contradict previous findings. We consider this to be due to the experimental conclusions obtained under different experimental conditions. The cell cycle time of ASMCs is 48–52 hours [28], and previous studies have focused on the effect of VEGF on the proliferation of ASMCs. However, this study focused on the impact of VEGF on the migration function of ASMCs. Our aim was to evaluate the impact of VEGF on experimental results within the measured migration time (24 hours). Our experimental time (24 hours) was shorter than the ASMC cell cycle (48–52 hours). Our findings are based on the conclusions drawn under the above experimental conditions. VEGF-induced ASMC migration is related to cytoskeletal remodeling. Cell migration is usually accompanied by reconstruction of the cytoskeleton, and actin is a component of the cytoskeleton. Actin exists in cells in two groups of free and polymerized states. The free state of actin is called G-actin, and the polymerized state of actin is called F-actin [29]. Under certain conditions, the dissociated actin and polymerized actin are in equilibrium. When stimulated by external chemical or physical signals, the free G-actin monomer in the cell will combine to form F-actin in the polymerized state and then form actin filaments through its own spiral, which is called cytoskeletal remodeling [30, 31]. Phalloidin specifically binds with polymerized actin but not with free actin monomers. Under a confocal laser scanning microscope, fluorescence-labeled phalloidin clearly displayed the morphology and distribution of the cytoskeleton. In this experiment, after VEGF stimulation of ASMCs, the cytoskeleton labeled with phalloidin was observed under a confocal laser scanning microscope. The cell actin filaments increased, polarized, pseudopodia were prominent, and the cytoskeleton was reconstructed. However, after adding the Rho kinase inhibitor Y27632, the cell contour changed, the actin filament decreased, the distribution was disordered, there were no cell pseudopodia, and cytoskeletal remodeling was significantly inhibited. The experiment suggested that VEGF induced ASMCs to promote migration by inducing cytoskeletal remodeling.

VEGF-induced ASMC migration is related to the activation of the cell RhoA/ROCK pathway. The RhoA/ROCK signaling pathway is involved in the regulation of cell morphology, adhesion, proliferation and migration [32]. Many previous studies have reported that the Rho family of GTPases plays an important role in regulating cell migration [21, 33]. RhoA is a member of the small GTPase Rho family. RhoA binds GTP to activate the downstream protein ROCK, which in turn phosphorylates downstream ROCK substrates. Activation of the RhoA/ROCK pathway in ASMCs upregulates the phosphorylation of MLC, providing power for cell migration. Actin reconstructs the cytoskeleton through polymerization and depolymerization. Myosin has adenosine triphosphatease (ATPase) activity. When myosin interacts with actin, myosin can hydrolyze ATP and convert chemical energy into mechanical force, and myosin pulls actin filaments to produce contractile force, providing power for cell migration [34].

MLC phosphorylation is a necessary condition to initiate actin contractile cell migration [35]. After the cells receive external stimulation signal activation, intracellular calmodulin upregulates the concentration of Ca^2+^, activates MLC kinase (MLCK), and upregulates the level of p-MLC [36]. MLC phosphatase (MLCP) catalyzes the dephosphorylation of p-MLC. MYPT_1_ is the effector of ROCK, and the level of p-MYPT_1_ is an important indicator of ROCK activity [37]. MYPT_1_ has two inhibitory phosphorylation sites, T696 and T853, and phosphorylation of these sites inhibits the phosphatase activity of the whole enzyme. ROCK is the protein that catalyzes the phosphorylation of MYPT_1_ at T696 or T853, and p-MYPT_1_ inhibits the activity of MLCP, thus inhibiting the dephosphorylation of MLC [38]. The level of p-MLC is the result of the dynamic regulation and balance of kinases and phosphatases. To explore the expression patterns of RhoA/ROCK downstream proteins at different times after VEGF-induced ASMCs, we used Western blotting to detect the expression levels of marker proteins at 0, 3, 6, 12, and 24 hours. VEGF induced increases in the expression levels of ROCK_1_, p-MYPT_1_, and p-MLC in ASMCs. We also found that after 0 to 6 hours of stimulation, the expression levels of ROCK_1_, p-MYPT_1_, and p-MLC showed an upward trend. These data also suggest that the early addition of VEGF has the best effect on promoting migration in ASMCs. Y27632 is a specific inhibitor of ROCK protein kinase, and it inhibits the activity of ROCK_1/2_ by competitively binding to ATP [39]. Our study showed that in the VEGF-induced ASMC migration experiment, adding the inhibitor Y27632 inhibited the activation of ROCK, the signal transduction of the RhoA/ROCK pathway was inhibited, the downstream substrate p-MYPT_1_ expression was reduced, MLCP catalyzed the hydrolysis of p-MLC, and downregulated the level of p-MLC. These results show that Y27632 can inhibit ASMC migration by inhibiting the RhoA/ROCK signaling pathway.

However, our research has some limitations. First, all the cells used in our current research were obtained from in vitro experiments on primary ASMCs of healthy people. Second, our research has not been further verified in an animal asthma model, and the application of relevant evidence to the treatment of allergic asthma is still limited. In addition, VEGF participates in airway smooth muscle remodeling and can induce an asthma-like phenotype, but the RhoA/ROCK pathway may not be the only signaling pathway, and other signaling pathways or targets need further study. Finally, Y27632 is an inhibitor of ROCK and Y27632 was used to study the signaling pathway in this experiment, but the single Y27632 group was not shown in this study, perhaps this is also one of the limitations of this study.

In summary, VEGF is playing an increasingly important role in the research of airway diseases and is considered a potential pro-remodeling factor. This study demonstrated that VEGF activates the RhoA/ROCK pathway, induces F-actin reorganization, and improves the migration of ASMCs. In addition, the ROCK inhibitor Y27632 abolished downstream effects. In addition to increasing lung inflammation sensitization, tissue edema and vascular remodeling in asthma patients, VEGF may participate in airway remodeling by inducing the migration of local smooth muscle. Therefore, strategies targeting VEGF or signaling components may additionally be considered for trials in airway remodeling.

### Supplementary Information

**Additional file 1.**

**Additional file 2.**

## Data Availability

The data used to support the findings of this study are available from the corresponding author upon request.

## Abbreviations

VEGF: Vascular endothelial growth factor
ASMCs: Airway smooth muscle cells
MYPT_1_: Myosin phosphatase targeting subunit-1
MLC: Myosin light chain
MHC: Myosin heavy chain
MLCK: Myosin light chain kinase
MLCP: Myosin light chain phosphatase
p-MYPT_1_: Phosphorylation of myosin phosphatase targeting subunit-1
p-MLC: Phosphorylation of myosin light chain
Rho: Ras homolog; ROCK: RhoA-associated kinase
RhoA: Ras homolog protein A
VEGFR1: Vascular endothelial growth factor receptor 1
CCK-8: Cell Counting Kit-8
PI3K: Phosphoinositide 3-kinase
GTP: Guanosine triphosphate
FBS: Fetal bovine serum
PBS: Phosphate buffered sodium
OD: Optical density
BCA: Bicinchoninic acid
PVDF: Polyvinylidene difluoride
PDGF: Platelet-derived growth factor
TGF-β: Transforming growth factor-β
PGD2: Leukotriene, prostaglandin D2
IL-8: Interleukin-8
TSLP: Thymic stromal lymphopoietin
GDIs: Guanine nucleoside dissociation inhibitors
GAPs: GTPase-activating proteins
GEFs: Guanine nucleoside exchange factors
