# Supplementary material for: Vascular endothelial growth factor induces the migration of human airway smooth muscle cells by activating the RhoA/ROCK pathway
Source: BMC Pulm Med. 2023 Dec 13;23:505. doi: 10.1186/s12890-023-02803-y (PMC10720058; doi:10.1186/s12890-023-02803-y)
Supplement: Supplementary file 1 — Additional file 1. [file 12890_2023_2803_MOESM1_ESM.pdf]

Figure 5 (A)

Images with different exposures

$ROCK_1$

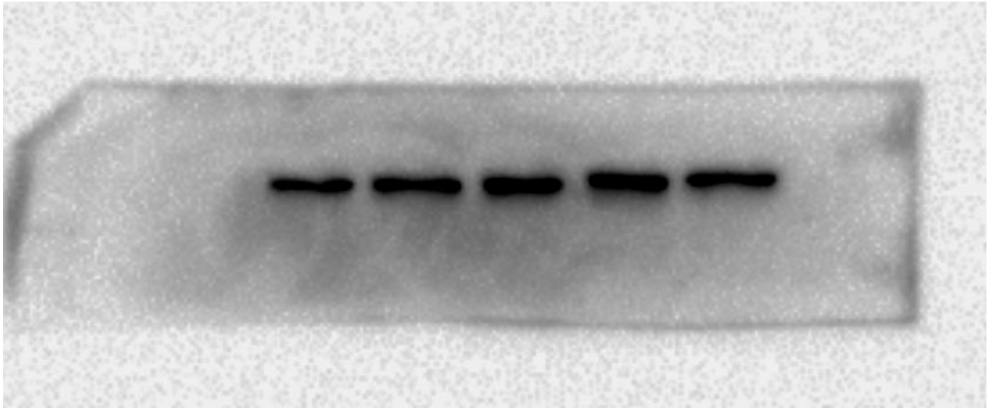

160KD

$ROCK_1$

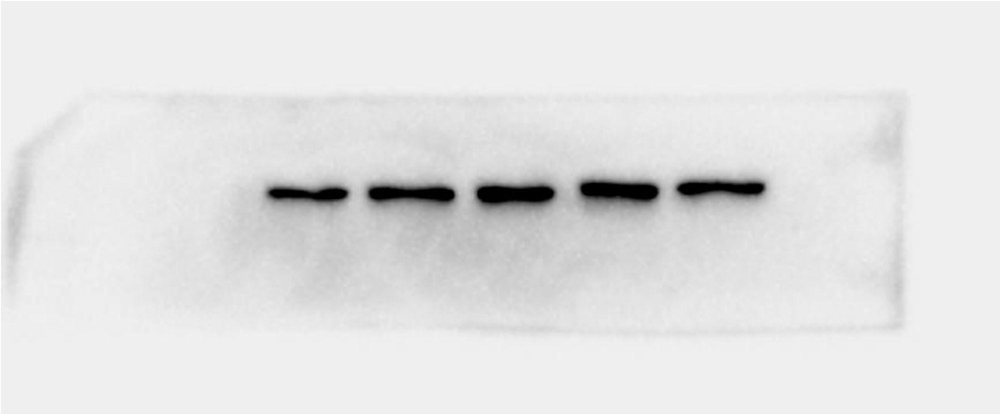

160KD

Figure 5 (A)

Images with different exposures

β-actin

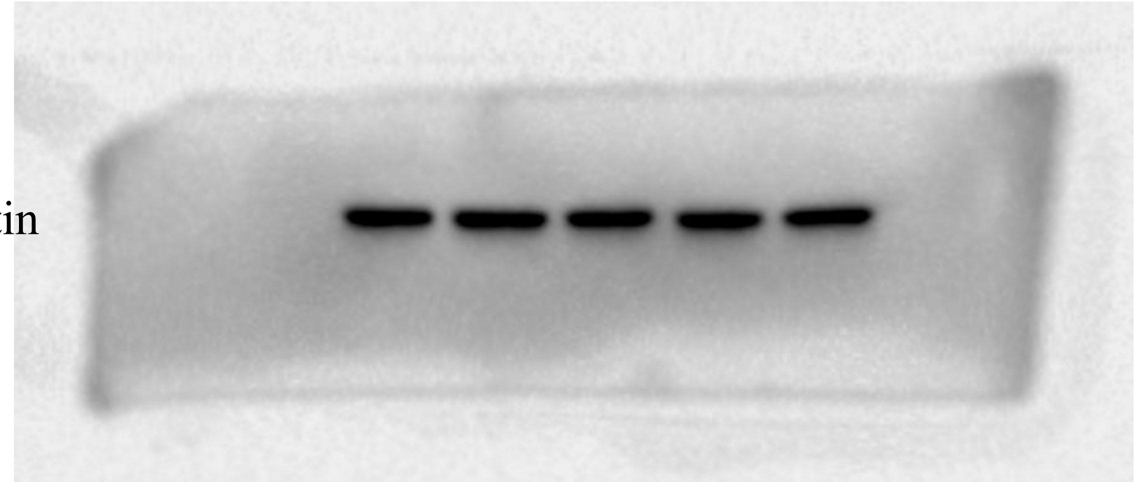

42KD

β-actin

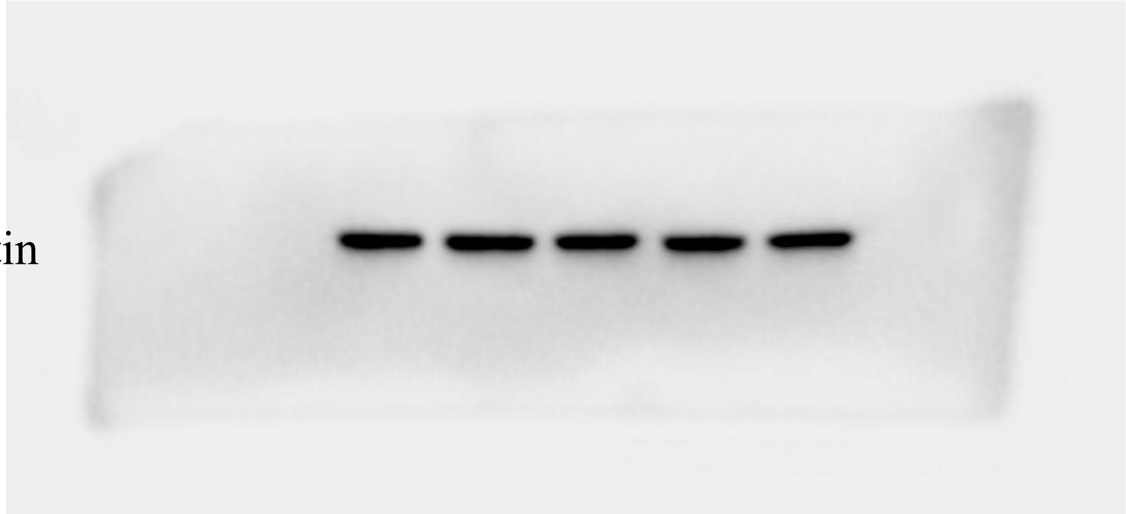

42KD

Figure 5 (B)

Images with different exposures

$ROCK_1$

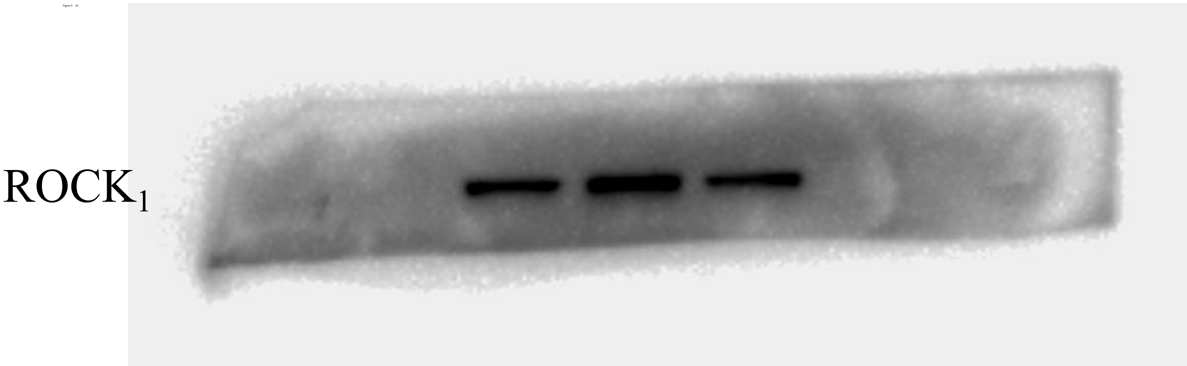

160KD

$ROCK_1$

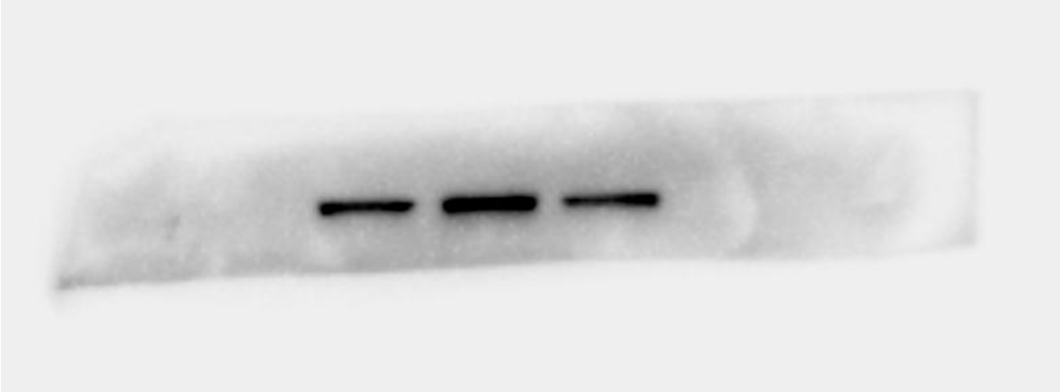

160KD

Figure 5 (B)

Images with different exposures

β-actin

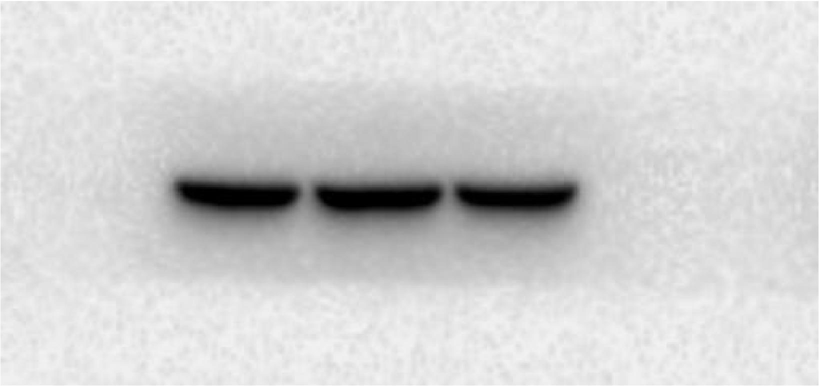

42KD

β-actin

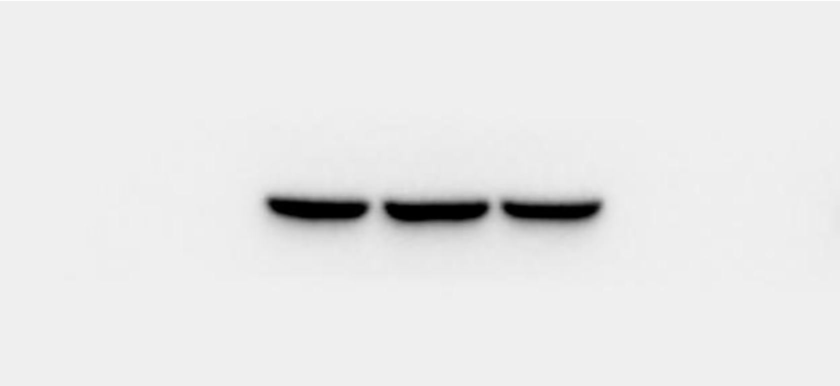

42KD

Figure 5 (C)

Images with different exposures

$p\text{-}MYPT_1$ 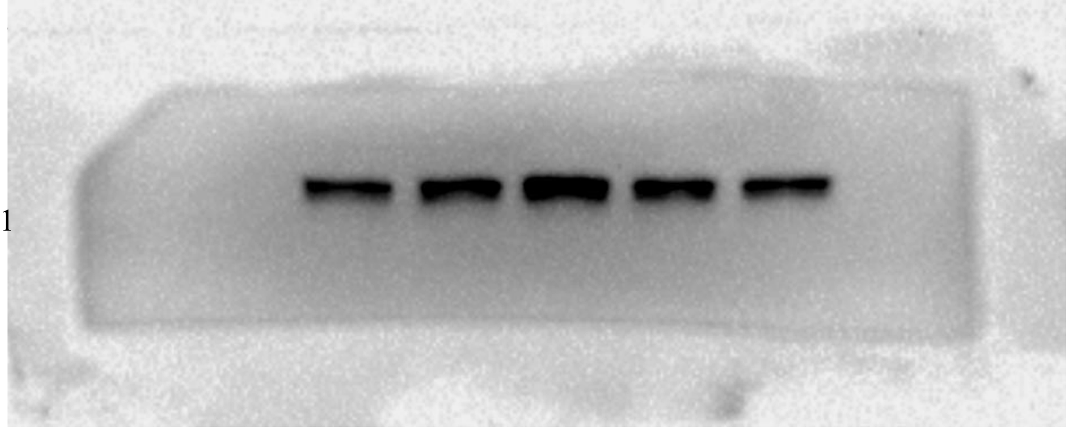 140KD

$p\text{-}MYPT_1$ 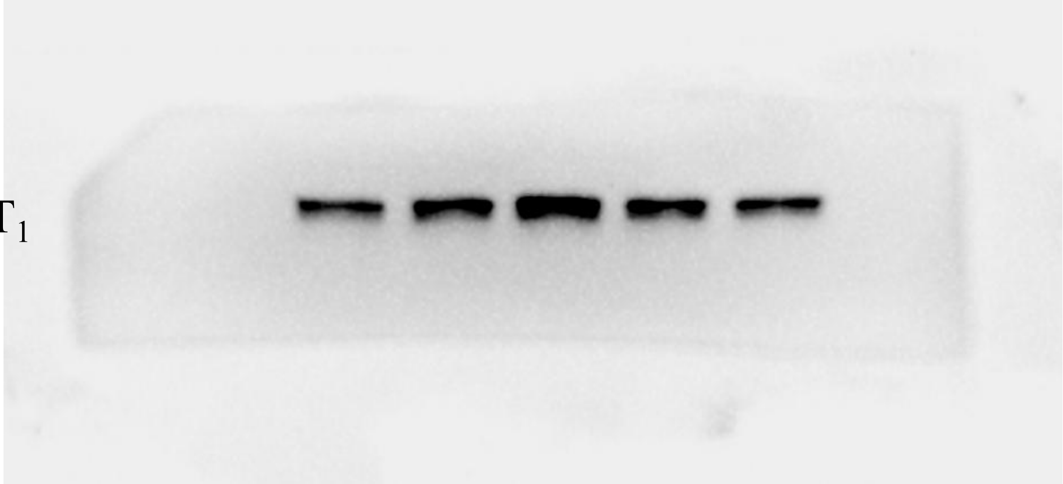 140KD

Figure 5 (C)

Images with different exposures

MYPT$_1$ 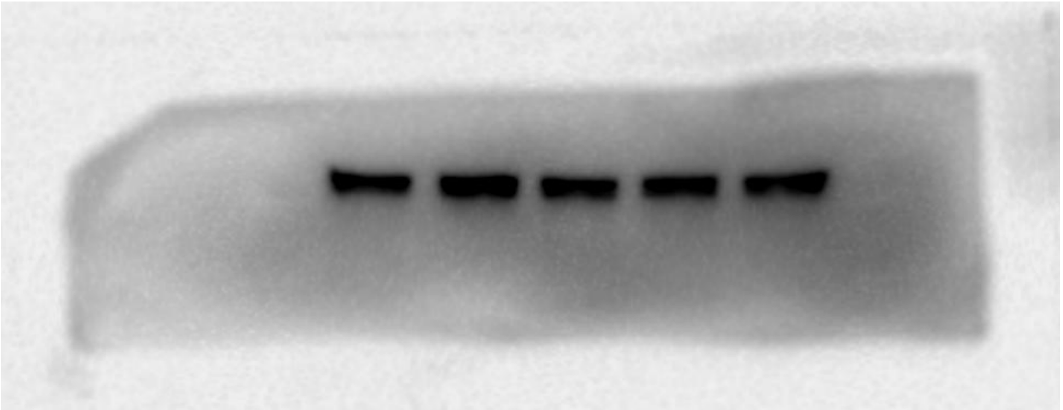 140KD

MYPT$_1$ 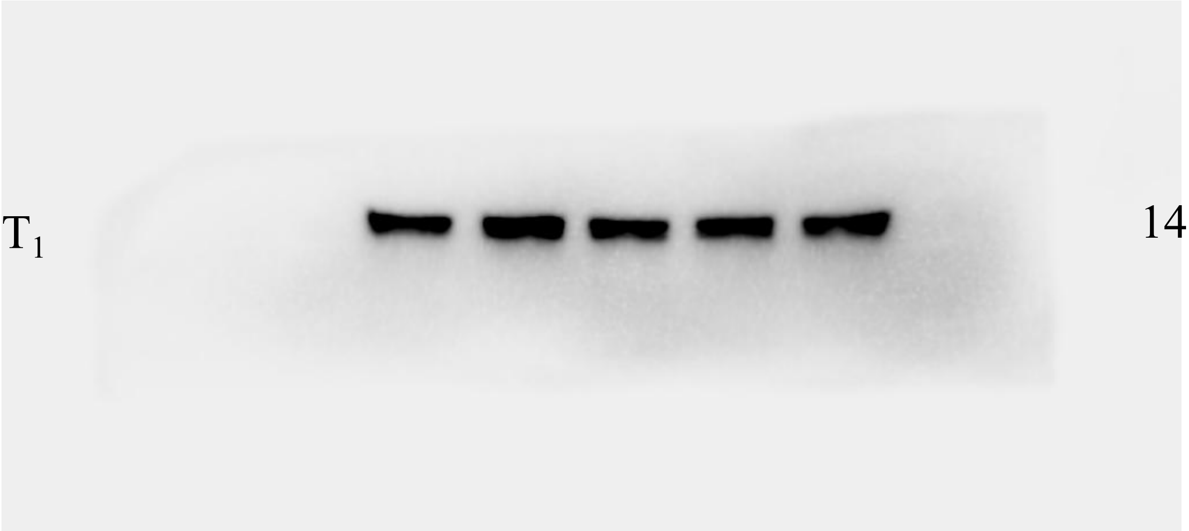 140KD

Figure 5 (C)

Images with different exposures

β-actin

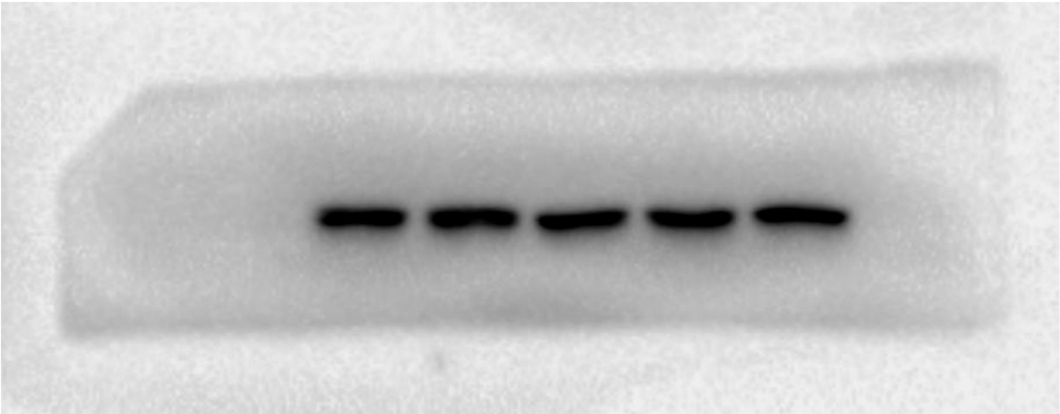

42KD

β-actin

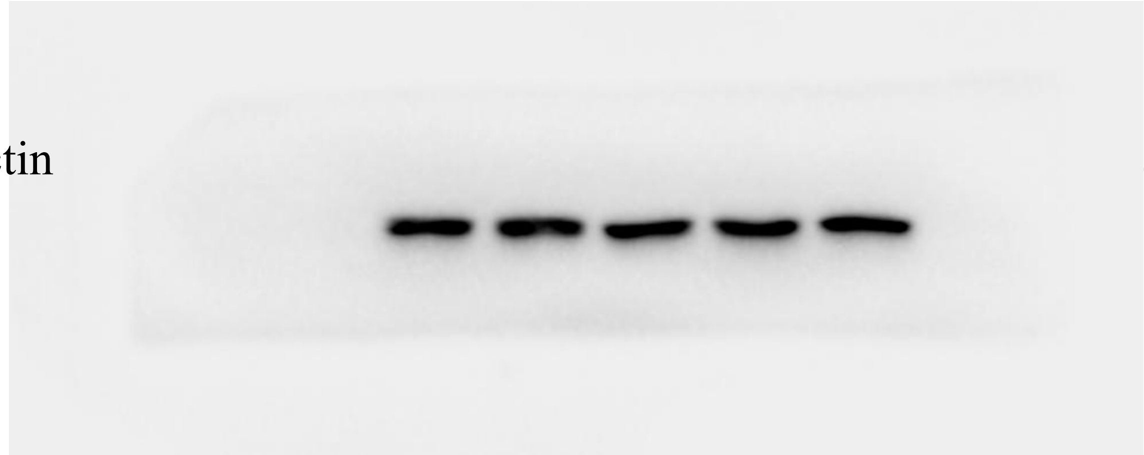

42KD

Figure 5 (D)

Images with different exposures

p-$MYPT_1$

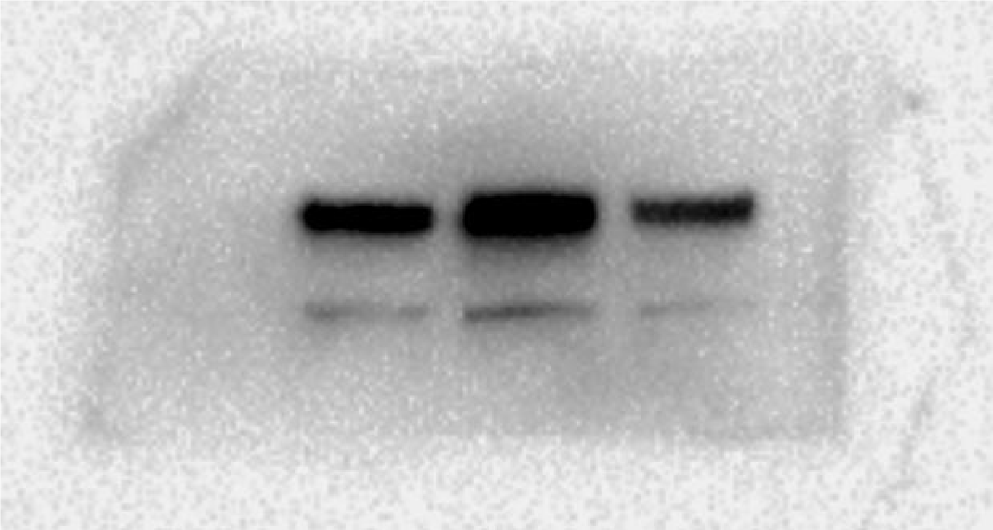

140KD

p-$MYPT_1$

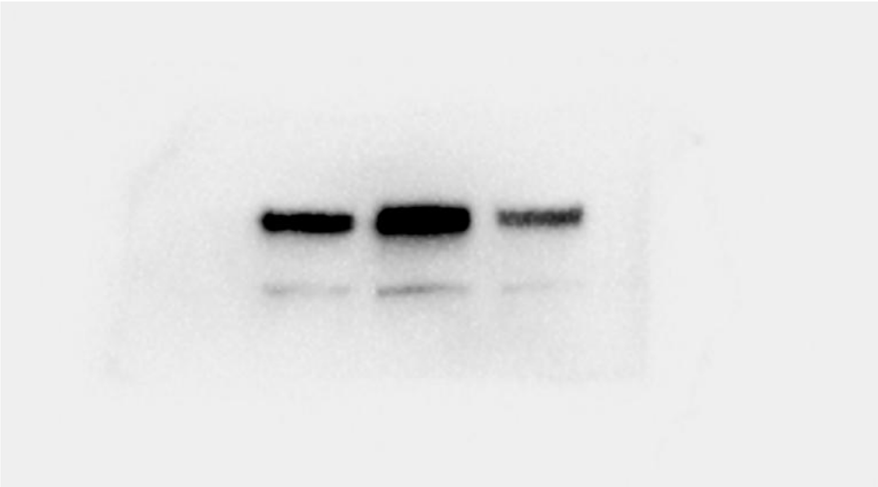

140KD

Figure 5 (D)

Images with different exposures

MYPT$_1$

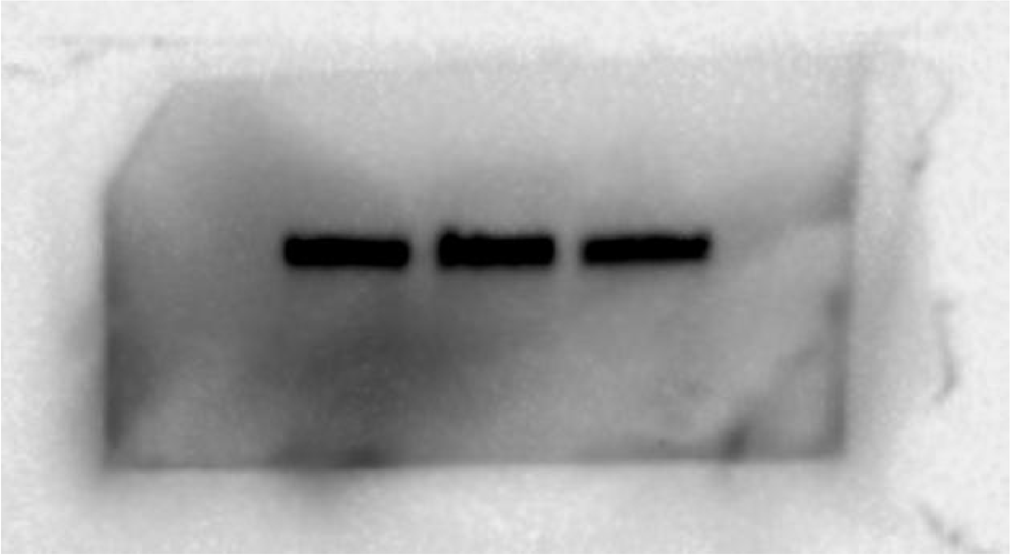

140KD

MYPT$_1$

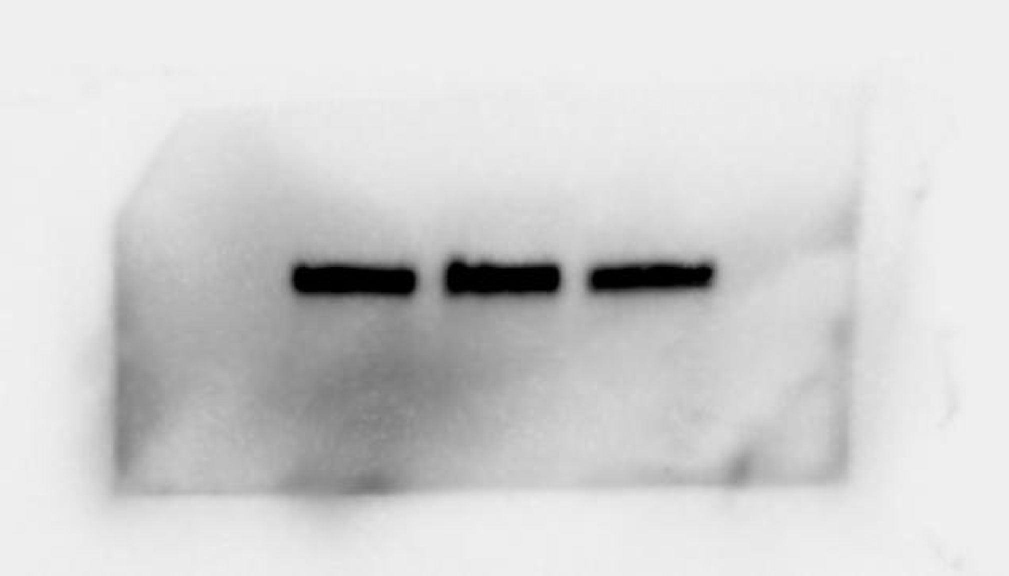

140KD

Figure 5 (D)

Images with different exposures

β-actin

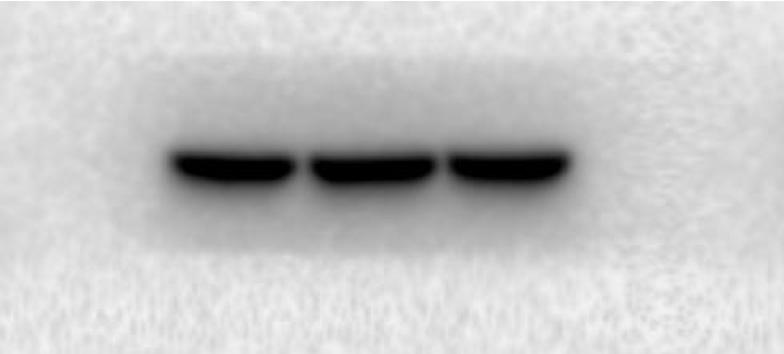

42KD

β-actin

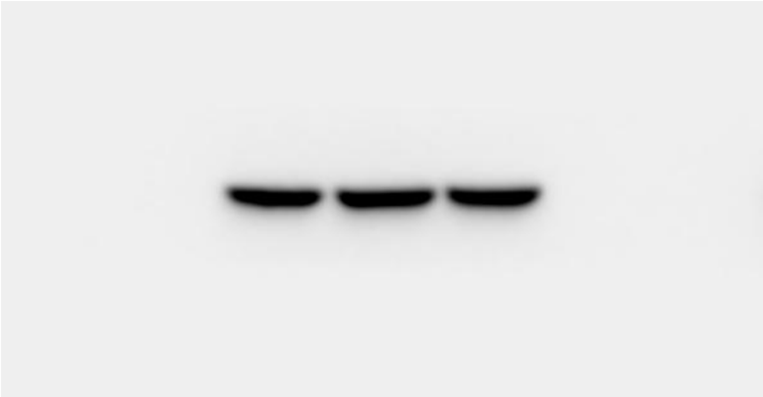

42KD

Figure 5 (E)

Images with different exposures

p-MLC

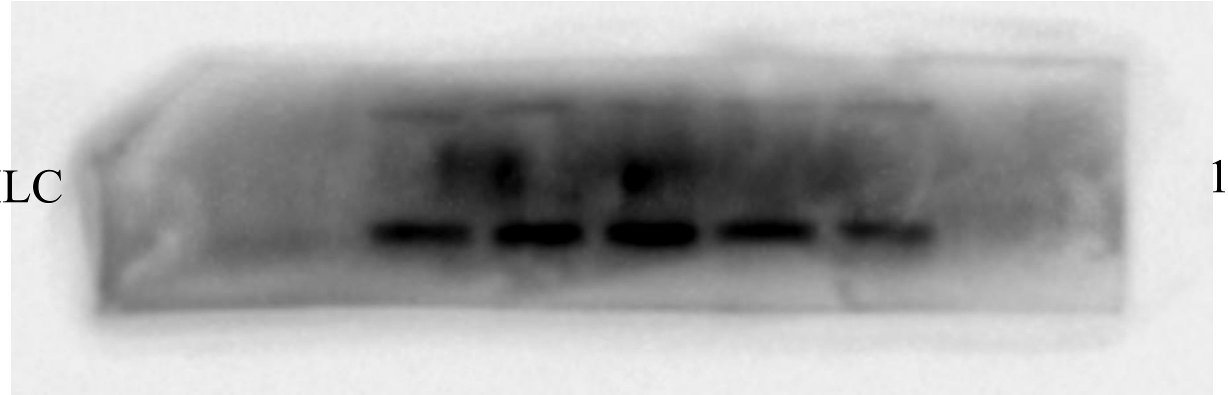

18KD

p-MLC

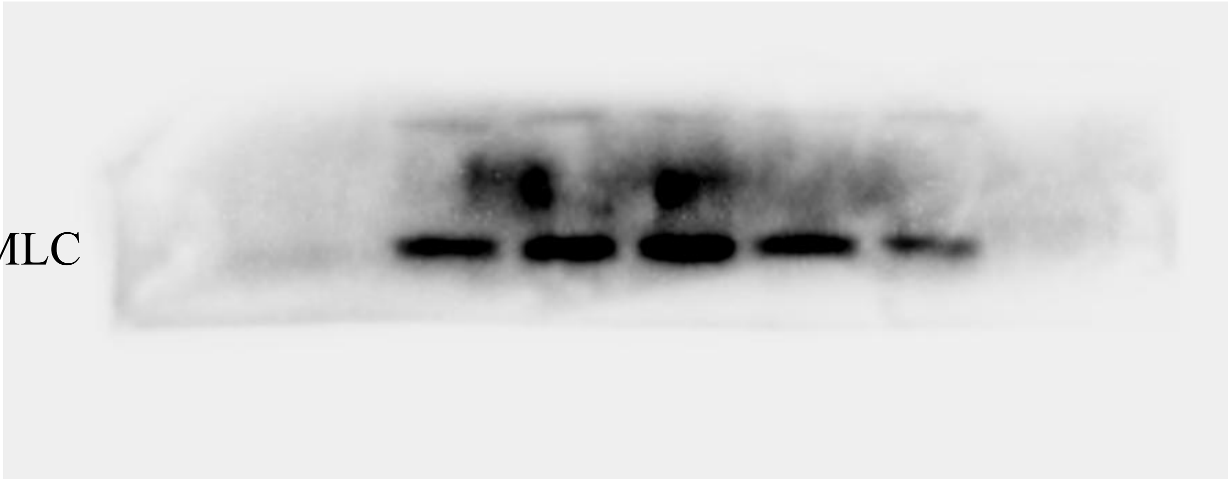

18KD

Figure 5 (E)

Images with different exposures

MLC

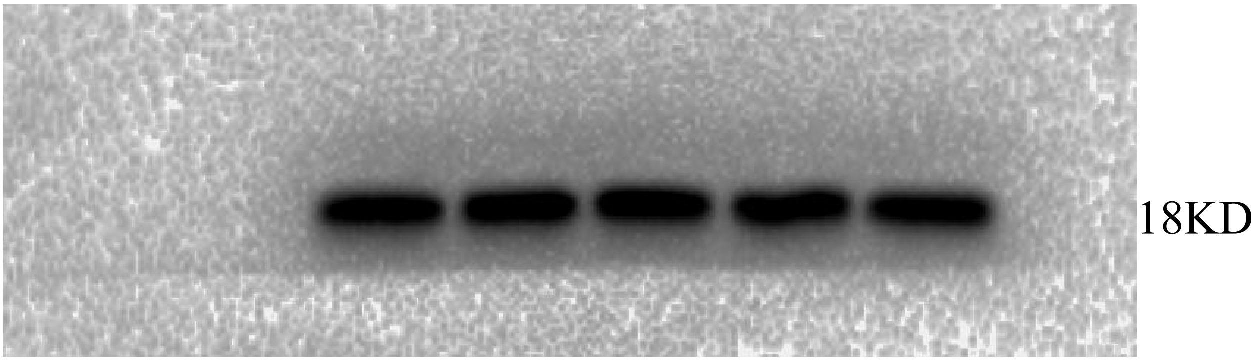

18KD

MLC

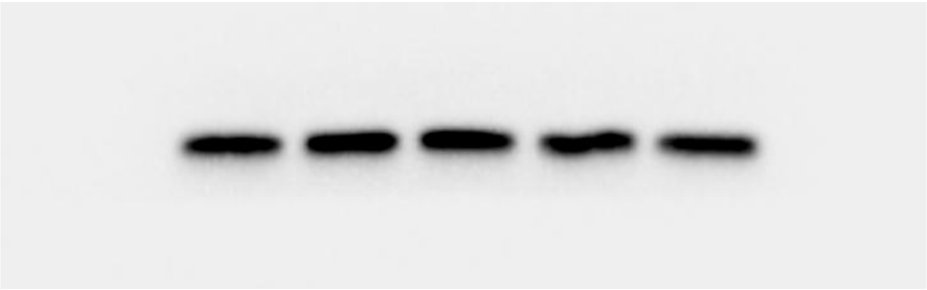

18KD

Figure 5 (E)

Images with different exposures

β-actin

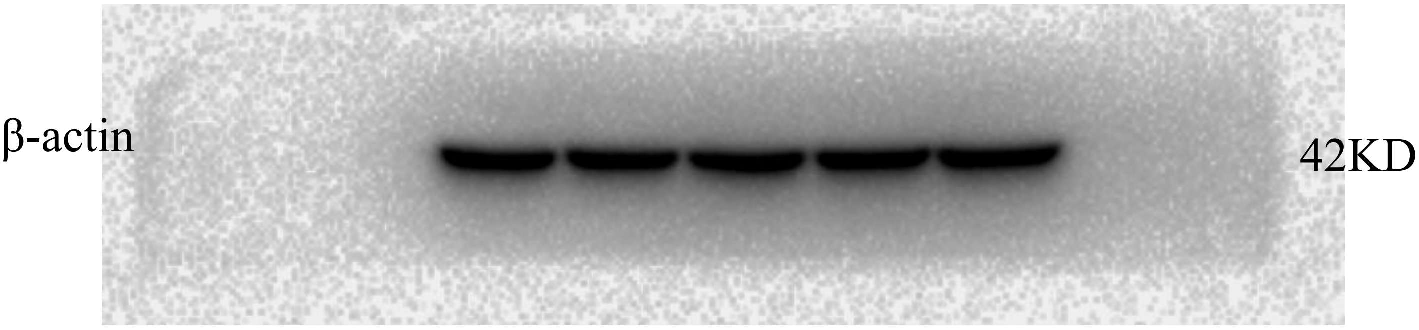

42KD

β-actin

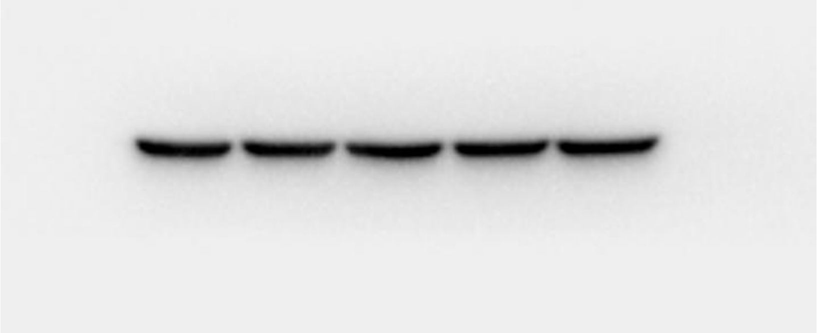

42KD

Figure 5 (F)

Images with different exposures

p-MLC

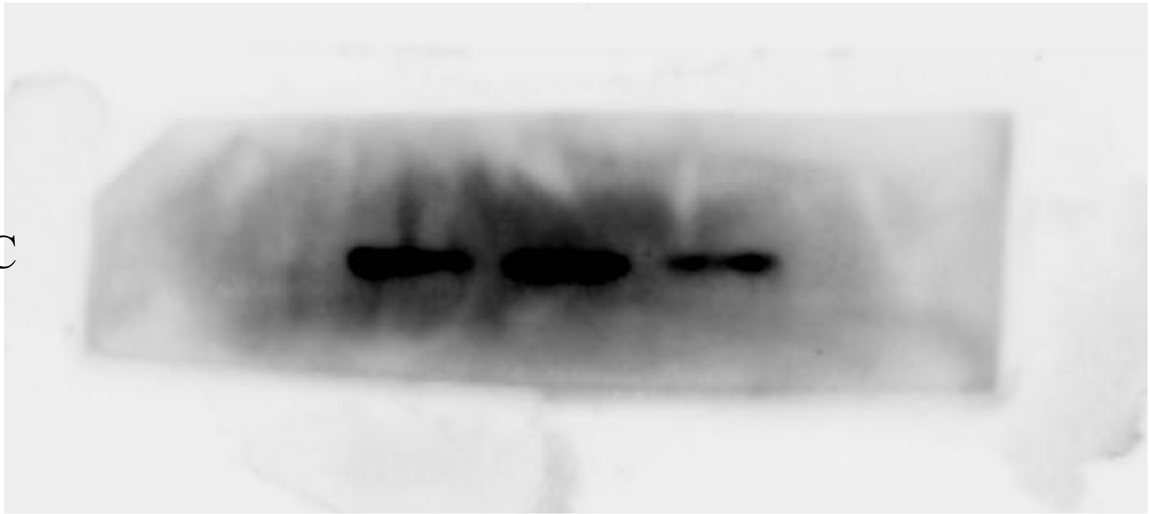

18KD

p-MLC

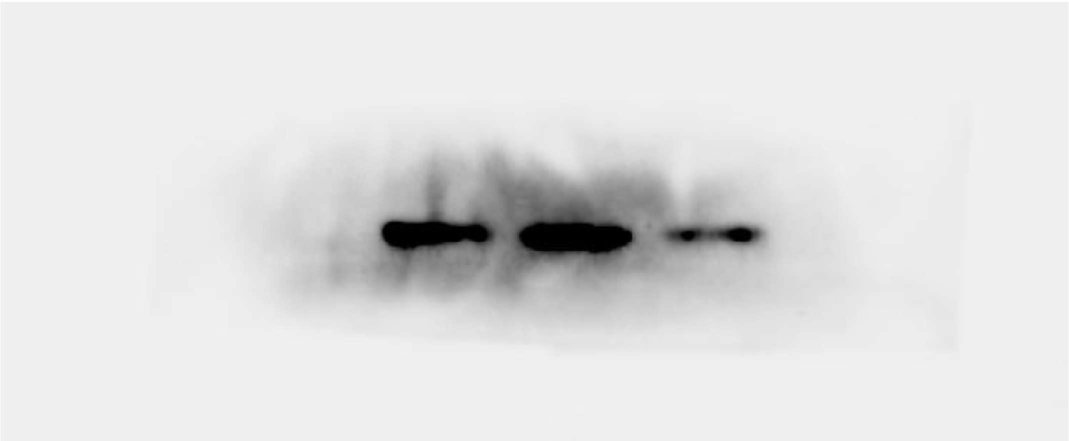

18KD

Figure 5 (F)

Images with different exposures

MLC 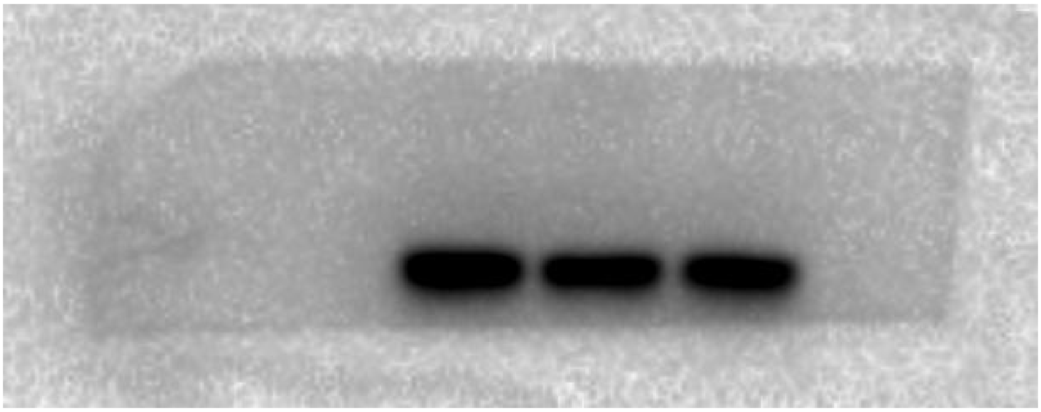 18KD

MLC 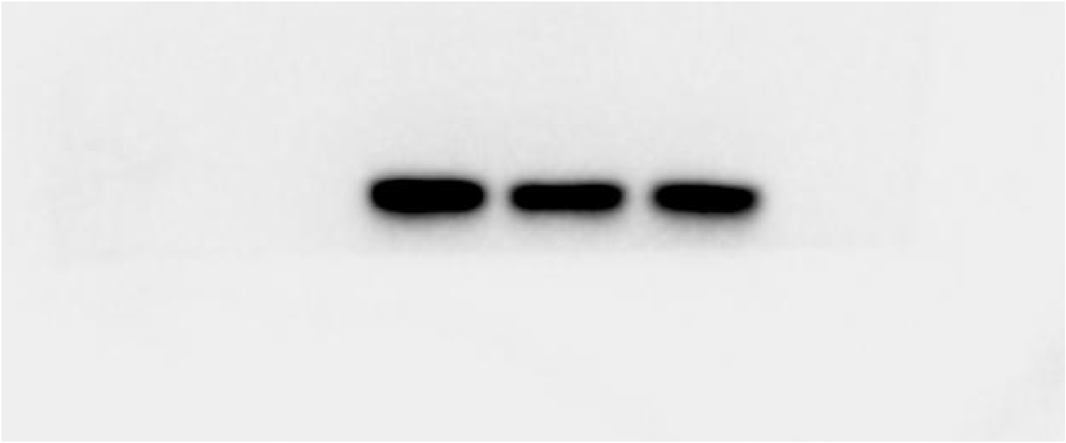 18KD

Figure 5 (F)

Images with different exposures

β-actin

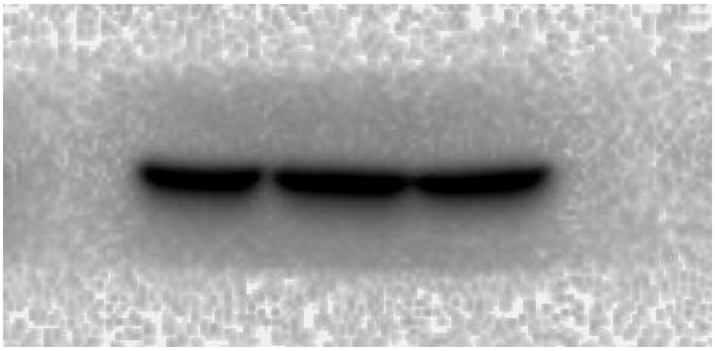

42KD

β-actin

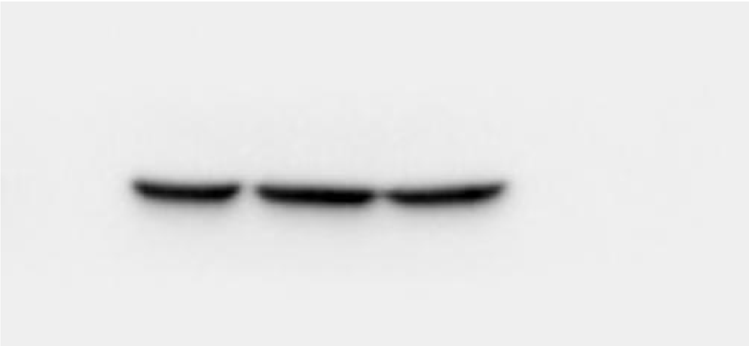

42KD
